# CFTR protects against vascular inflammation and atherogenesis in apolipoprotein E-deficient mice

**DOI:** 10.1042/BSR20170680

**Published:** 2017-07-07

**Authors:** Zhengzhang Li, Zhe Shen, Haoping Xue, Shi Cheng, Qun Ji, Yutan Liu, Xiangjun Yang

**Affiliations:** Department of Cardiology, The First Affiliated Hospital of Soochow University, Suzhou, Jiangxu 215006, China. 2Department of Cardiology, Gaoyou People's Hospital, Gaoyou, Jiangsu 225600, China

**Keywords:** atherosclerosis, CFTR, inflammation, macrophages, MAPKs, NFκB

## Abstract

Atherosclerosis is a chronic inflammatory disease of the vascular wall. Dysfunction of cystic fibrosis transmembrane conductance regulator (CFTR) has been shown to result in inflammatory responses in cystic fibrosis (CF) patients. However, little is known about the role of CFTR in vascular inflammation and atherogenesis. Our results showed that CFTR was dominantly expressed in macrophages of atherosclerotic plaque and reduced in aorta and aortic sinus from atherosclerotic apolipoprotein E-deficient (apoE^−/−^) mice. *In vivo* administration of adenovirus encoding CFTR (Ad-CFTR) with apoE^−/−^ mice fed on high-fat diet (HFD) improved plaque stability by decreasing lipid accumulation and necrotic area and increasing smooth muscle cell content and collagen. The Ad-CFTR-treated mice also displayed reduced proinflammatory cytokines levels in aorta and peritoneal macrophages, whereas the anti-inflammatory M2 macrophage markers were increased. Confocal microscopy revealed that the infiltration of T lymphocytes, neutrophils, and macrophages in aortic sinus was markedly attenuated in Ad-CFTR-treated apoE^−/−^ mice. Moreover, *in vitro* experiments showed that overexpression of CFTR inhibited ox-LDL-induced the migration of peritoneal macrophages. Finally, it was observed that CFTR up-regulation suppressed NFκB and MAPKs activity induced by ox-LDL. Inhibition of JNK or ERK abrogated CFTR down-regulation induced NFκB activation, whereas NFκB inhibitor had no effect on JNK or ERK activation. Taken together, these results demonstrate that CFTR prevents inflammation and atherogenesis via inhibition of NFκB and MAPKs activation. Our data suggest that CFTR may present a potential therapeutic target for the treatment of vascular inflammation and development of atherosclerotic disease.

## Introduction

Atherosclerosis is a chronic inflammatory disease and the major underlying cause of cardiovascular events, including myocardial infarction, sudden cardiac death, and ischemic stroke [1–3]. Inflammation is an essential contributor during the development of atherosclerosis [4]. It initiates the infiltration of monocytes as well as monocyte-derived macrophages into the subendothelial area of vessels [4,5]. The accumulation of macrophages in the arterial wall correlates with the extent of foam cell formation under the condition of modified low-density lipoprotein, which in turn leads to the development of atherosclerotic lesions [6,7]. When excessive foam cells undergo apoptosis or necrosis, it leads to plaque instability and rupture, resulting in various cardiovascular events [3,8]. Hence, understating the molecular mechanisms that regulate the inflammatory responses could be a valuable strategy for the prevention and treatment of atherosclerosis.

Cystic fibrosis (CF) is a common autosomal recessive disease, which is caused by mutations in the gene encoding an anion channel, CF transmembrane conductance regulator (CFTR) [9]. Dysfunction or mutation of CFTR is associated with CF-related morbidities, including gastrointestinal abnormalities, pulmonary disease, and pancreatic insufficiency [10]. However, direct effects of CFTR on cardiovascular physiology are poorly understood. Increasing evidence have demonstrated the critical role of CFTR in the regulation of inflammation. CF patients are characterized by chronic airway inflammation with excessive infiltration of neutrophils and production of inflammatory cytokines [9,11,12]. In animal model, deficiency of CFTR exaggerated LPS-induced lung inflammation and injury by facilitating neutrophil recruitment, platelet aggregation, and thrombocytopenia [13–15]. Moreover, studies also have shown that CFTR expressed by macrophages altered bactericidal activity and regulated acute proinflammatory responses [16,17]. Despite the above findings, a detailed physiologic role of CFTR in monocytes/macrophages inflammation during atherosclerosis remains largely elusive. Therefore, in the present study, we investigated whether CFTR is causally involved in atherosclerosis-related inflammation as well as atherogenesis and if so, to clarify its mechanism in these processes.

## Materials and methods

### Construction of CFTR adenovirus

The adenovirus encoding CFTR (Ad-CFTR) was generated by ViraPower Adenoviral Expression System (Invitrogen, NY, U.S.A.). The wild-type full-length mouse CFTR was cloned into pAd/CMV/V5-DEST Gateway vector (Invitrogen). The vector was linearized by standard cesium chloride/Ethidium Bromide equilibrium density gradient centrifugation. Linear DNA was transfected into QBI-293 cells at 50–70% confluence. The recombinant adenovirus was packaged and amplified in 293A cells and purified by Fast Trap Adenovirus Purification and Concentration Kit (Millipore, MA, U.S.A.). The virus titer was determined by absorbance at 260 nm. The adenovirus expressing LacZ alone (Ad-LacZ) was obtained from Invitrogen and used as a negative control.

### Animal model

At 8 weeks, male apolipoprotein E-deficient (apoE^−/−^) mice (Jackson Laboratory) were subjected to either a normal diet (ND) or a high-fat diet (HFD, 60% of calories from fat, D12492, Research Diets, Inc., NJ, U.S.A.) for 12 weeks. Simultaneously, HFD-treated mice were injected with 5.0 × 10^10^ vp of Ad-CFTR or Ad-LacZ via tail vein. The adenovirus injection was repeated weekly for 8 weeks. All animals were killed 8 weeks after the first injection. Mice were housed in an animal facility at Soochow University with free access to water *ad libitum* and a 12-h light-dark cycle at 23 ± 1°C. The present study was approved by the Institutional Animal Care and Use Committee of Soochow University and carried out in accordance with the Guide for the Care and Use of Laboratory Animals of Soochow University.

### Cell culture and treatment

Thioglycollate-elicited peritoneal macrophages were collected from apoE^−/−^ mice by peritoneal washing with ice-cold, serum-free RPMI1640 (Invitrogen). After adherence for 1  h at 37°C and 5% CO_2_, the medium was replaced by fresh RPMI1640 containing 10% FBS, 2 mM glutamine, 50 U/ml penicillin, and 50 μg/ml streptomycin (all from Invitrogen).

Mouse aortic smooth muscle cells (MASMCs) were isolated as previously described [18]. After carefully removing the intima and adventitia of the mouse thoracic aortas, tissues were cultured in Dulbecco’s modified Eagle’s medium (DMEM) (Invitrogen), supplemented with 10% FBS, 50 U/ml penicillin, and 50 μg/ml streptomycin. Cells migrating from explants were collected and maintained in growth medium. MASMCs at passages 2–3 were used for experiments.

Mouse aortic endothelial cells (MAECs) were isolated from the thoracic aortas as recently described [19]. Briefly, the vessels were opened longitudinally and cut into 3-mm pieces and plated on a matrigel precoated dish. The pieces were incubated in DMEM/F-12 medium with 10% FBS, 50 U/ml penicillin, 50 μg/ml streptomycin, 10 μg/ml heparin sodium, and 5 ng/ml basic fibroblast growth factor (Invitrogen). After 3–4 days, the tissues were removed and the cells were harvested. Cells from passages 2–3 were collected for analysis.

For an *in vitro* study, Ad-CFTR infection in primary mouse peritoneal macrophages was performed at 50 multiplicity of infection (MOI) in RPMI1640 containing 2% FBS for 24 h. The cells were pretreated with Ad-CFTR or CFTRinh-172 (10 μM) (Selleckchem, TX, U.S.A.) for 24 h, and then stimulated with ox-LDL (80 μg/ml, Yiyuan Biotechnology, Guangzhou, China) for another 24 h in the presence or absence of PD98059 (10 μM), SP600125 (10 μM), or BAY11 (20 μM) (all from Sigma, MO, U.S.A.).

### Western blotting

Protein was extracted from the aorta or peritoneal macrophages in RIPA lysis buffer with protease inhibitor cocktail (Thermo, MA, U.S.A.). Nuclear proteins were extracted using a Nuclear/Cytosol Fractionation Kit (BioVision, CA, U.S.A.) according to the manufacturer’s instructions. Equal content of protein was used for SDS/PAGE, and then transferred on to PVDF membranes (Millipore). The membranes were respectively probed with the following specific antibodies: CFTR (1:500), IL-1β (1:1000), IL-6 (1:500), IL-10 (1:600), and Arg (1:1000) (Santa Cruz, CA, U.S.A.); p65 (1:800), Lamin B (1:1000), p-JNK (1:500), JNK (1:1000), p-p38 (1:1000), p38 (1:1000), p-ERK (1:500), and β-actin (1:2000) (Cell Signaling Technology, MA, U.S.A.). After incubation with appropriate horseradish peroxidase (HRP)–conjugated secondary antibodies (1:1000) (Cell Signaling Technology), the target band was detected with enhanced chemiluminescent HRP substrate (Millipore).

### Quantitation of atherosclerotic lesions

The entire aorta was carefully isolated from mouse and opened longitudinally. The aortic roots were embedded in Tissue-Tek® (Sakura, CA, U.S.A.) and 5-µm frozen sections were collected every 50 μm in eight different littermates from each group. The atherosclerotic lesion in the entire aorta and aortic sinus was assessed by Oil Red O (Sigma) and quantitated using ImagePro Plus 6.0 software (Media Cybernetics, MD, U.S.A.) as recently described [7].

### Immunofluorescence

For immunofluorescence staining, sections were incubated with 3% H_2_O_2_ for 15 min at room temperature and then blocked in 10% goat serum diluted with PBS for 1 h. After three washings with PBS for 5 min, sections were incubated with primary antibodies against CFTR (1:50), α-SMA (1:100) (Santa Cruz), MOMA-2 (1:100), CD3 (1:50), and Ly-6G (1:100) (Abcam, MA, U.S.A.) overnight at 4°C, followed by incubation with appropriate secondary antibodies for 1 h at room temperature. The following secondary antibodies were used: FITC-labeled donkey anti-goat (1:100) and cy3-labeled donkey anti-rabbit (1:100) (Boster, Wuhan, China). To examine the apoptotic cells in atherosclerotic plaque, the sections of aortic sinus were incubated with FITC-dUTP (Roche, NY, U.S.A.) for 1 h at 37°C. Nuclei were counterstained with DAPI. The sections were analyzed by a confocal microscope (LSM5, Zeiss, München, Germany) with 400× magnification. Staining intensity was quantitated using ImagePro Plus 6.0 software.

### PicroSirius Red staining

For immunohistological staining of collagen, frozen sections of aortic sinus were fixed with 10% buffered formalin for 10 min. After rinsing with distilled water, the sections were incubated with 0.1% Sirius Red dye (Sigma) in saturated picric acid for 3 h. After rinsing with 0.01 N HCl and distilled water, sections were dehydrated in series of increasing concentrations of ethanol followed by xylene, and photographed with Zeiss LSM5 confocal microscope. Collagen content within the atherosclerotic lesion area was quantitated using ImagePro Plus 6.0 software.

### Flow cytometry

The polarization state of peritoneal macrophages was determined by flow cytometric analysis. Prior to staining, 1 × 10^6^ peritoneal macrophages were blocked with 1% BSA in 100 μl PBS at 4°C for 30 min. For intracellular staining, cells were fixed with fixation buffer and permeabilized with Permeabilization Wash Buffer (BioLegend, San Diego, CA, U.S.A.) followed by incubation with F4/80–conjugated with phycoerythrin (PE) (1:200) (Santa Cruz), iNOS–conjugated with allophycocyanin (APC) (1.0 μg/ml) (BioLegend), Arg1–conjugated with PerCP (1:200) (Santa Cruz). Macrophages were gated by forward and sideward scatter. Flow cytometry was performed using a FACSAria™ flow cytometer (BD Biosciences, San Jose, CA, U.S.A.) and data were analyzed using BD Flow Software.

### Macrophage migration assay

Cell migration was quantitated by use of 24-well Transwell inserts with polycarbonate filters (8-mm pore size) (Corning Costar, MA, U.S.A.). Mouse peritoneal macrophages were seeded on to the membranes of the upper chambers and infected with Ad-CFTR or Ad-LacZ for 24 h followed by ox-LDL incubation for another 24 h. After 48 h, the medium was changed to serum-free medium and the lower chambers of the inserts containing RPMI1640 with 10% FBS. After 3 h of migration, cells were fixed in methanol stained with 0.1% Crystal Violet. Macrophages found on the lower chambers were observed at magnification of 400× with a light microscope and quantitated using ImagePro Plus 6.0 software.

### Quantitative real-time PCR

Total RNA was isolated from the aorta tissues using TRIzol reagent (Takara, Dalian, China) according to manufacturer’s instructions. RNA (1 µg) was reverse-transcribed to cDNA using Transcriptor First Strand cDNA Synthesis Kit (Roche). Quantitative PCR was performed using SYBR Premix Ex Taq (Takara) and an ABI 7500 Real-Time PCR System (Applied Biosystems, CA, U.S.A.). The primer sequences for mouse MCP-1, TNF-α, IL-1β, IL-6, IL-10, Ym1, Mgl2, and Arg1 used were as follows: MCP-1, 5'-CCACTCACCTGCTGCTACTC-3' and 5'-AAGGCATCACAGTCCGAGTC-3'; TNF-α, 5'-GATCGGTCCCCAAAGGGATG-3' and 5'-GGGAGGCCATTTGGGAACTT-3'; IL-1β, 5'-ATCTCGCAGCAGCACATCAA-3' and 5'-ATGGGAACGTCACACACCAG-3'; IL-6, 5'-AGTTGCCTTCTTGGGACTGA-3' and 5'-TCCACGATTTCCCAGAGAAC-3'; IL-10, 5'-TGCACTACCAAAGCCACAAGG-3' and 5'-TGGGAAGTGGGTGCAGTTATTG-3'; Ym1, 5'-TCTCGAGGAAGCCCTCCTAA-3' and 5'-GTGGAAGTGAGTAGCAGCCT-3'; Mgl2, 5'-TGGAGCTTCCTGCTCATTCG-3' and 5'-CCCGCCGAATAATCTCTGGT-3'; Arg1, 5'-CCTTGTCGAATGGGCAGT-3' and 5'-CAGATATGCAGGGAGTCACC-3'; GAPDH, 5'-CCATCACCATCTTCCAGGAG-3' and 5'-GTGGTTCACACCCATCACAA-3'. Cycling conditions were as follows: 95°C for 5 min followed by 40 cycles of 95°C for 10 s, 55°C for 1 min, and 72°C for 30 s. The relative expression of each gene was calculated using the 2^−ΔΔ*C*^_T_ method and normalized against the internal control GAPDH.

### ELISA

The levels of MCP-1, IL-6, IL-10, and Arg1 in serum were measured by mouse MCP-1 ELISA Kit, IL-6 ELISA Kit, IL-10 ELISA Kit (Bosterbio, Wuhan, China), and Arg1 ELISA Kit (Cusabio, Wuhan, China) with the instructions provided by manufacturers. The absorbance was read using a microplate reader (Multiskan Spectrum, Thermo).

### Statistical analysis

Data were provided as mean value ± S.E.M., *n* represents the number of experiments. Comparisons were made amongst the groups and the two groups were analyzed by one-way ANOVA or Student’s two-tailed *t* test using SPSS10.0 statistical software (SPSS Inc., IL, U.S.A.). A probability value of <0.05 was considered statistically significant.

## Results

### Decreased expression of CFTR in mouse atherosclerotic plaque

**Figure 1:**
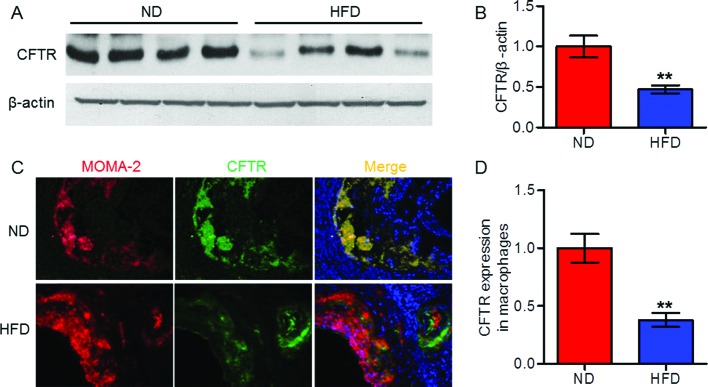
CFTR expression was decreased in atherosclerotic plaque of mice (**A**) After 12 weeks of HFD, Western blotting analysis for the expression of CFTR in aorta from ND-fed apoE^−/−^ mice and HFD-fed apoE^−/−^ mice. (**B**) Quantitation of CFTR protein expression. **P<0.01 compared with ND, *n*=10 in each group. (**C**) Representative double immunofluorescence staining of macrophages (red) and CFTR (green) in aortic sinus from HFD-fed apoE^−/−^ mice and ND-fed apoE^−/−^ mice (400× magnification). (**D**) Quantitation of CFTR distribution within macrophages was shown. **P<0.01 compared with ND, *n*=6 in each group.

To investigate the impact of atherosclerosis on CFTR expression in the vasculature, we analyzed CFTR level in mouse atherosclerotic plaque. CFTR protein expression in aorta from HFD-fed apoE^−/−^ mice was lower than that in ND-fed apoE^−/−^ mice (Figure 1A,B). Consistently, immunofluorescence staining revealed that CFTR expression in atherosclerotic lesion of HFD apoE^−/−^ mice was also decreased as compared with ND apoE^−/−^ mice. Furthermore, the decreased CFTR expression was found to be dominantly localized in MOMA-2-postive macrophages (Figure 1C,D). Nevertheless, CFTR was minimally expressed in MASMCs and MAECs. More importantly,* in vitro* study showed that ox-LDL treatment produced no effects on CFTR expression in VSMCs and endothelial cells (Supplementary Figure S1). The above results suggest that CFTR is down-regulated primarily in infiltrated macrophages of atherosclerotic plaque.

### Overexpression of CFTR reduced atherosclerosis progression in apoE^−/−^ mice

**Figure 2:**
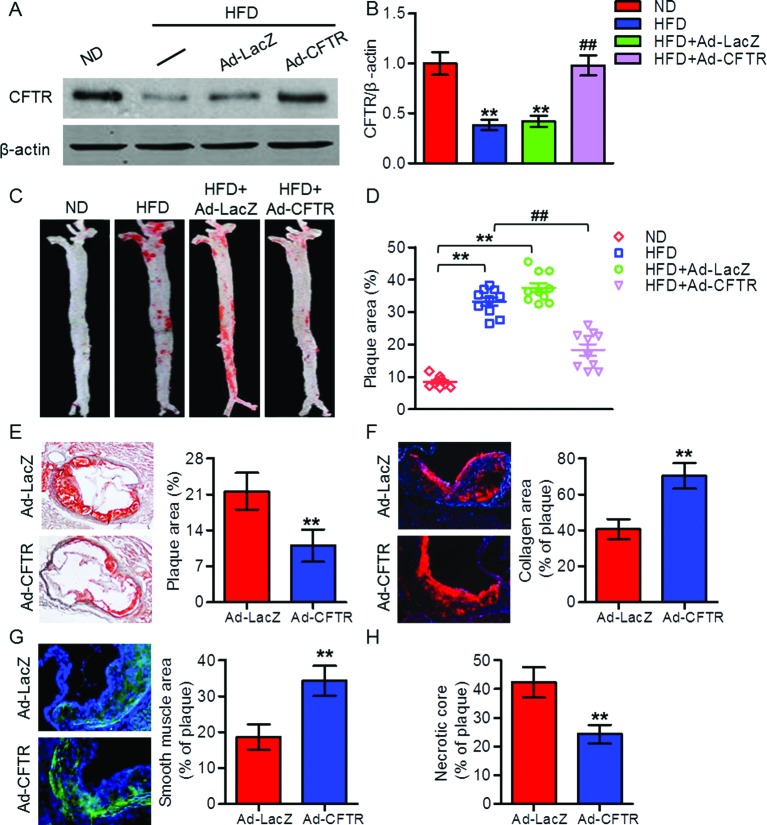
Effects of CFTR up-regulation on atherosclerotic lesions in apoE^−/−^ mice after HFD for 20 weeks (**A**) ApoE^−/−^ mice were administrated with an ND or an HFD for 12 weeks and then HFD-fed mice were injected with Ad-CFTR or Ad-LacZ via tail vein. After 8 weeks of adenovirus injection, aorta were isolated and subjected to Western blotting analysis for the detection of CFTR. (**B**) Quantitation of CFTR protein expression. **P<0.01 compared with ND; ^##^*P*<0.01 compared with HFD + Ad-LacZ, *n*=6 in each group. (**C**) Representative Oil red O staining of atherosclerotic lesions in aorta from HFD-fed apoE^−/−^ mice treated with Ad-LacZ or Ad-CFTR. (**D**) Quantitation of atherosclerotic plaque areas in aorta. **P<0.01 compared with ND; ^##^*P*<0.01 compared with HFD + Ad-LacZ, *n*=8 in each group. (**E–G**) Sections of aortic sinus were stained with Oil red O for atherosclerotic lesion area (E), PicroSirius Red for collagen (F), α-SMA for smooth muscle cells (G), and quantitation of the positive staining area was shown, respectively (400× magnification). (**H**) Quantitation of necrotic cores within aortic sinus of mice treated with Ad-LacZ or Ad-CFTR. **P<0.01 compared with HFD + Ad-LacZ, *n*=6 in each group.

To determine whether the decreased expression of CFTR was involved in the progression of atherosclerosis, apoE^−/−^ mice were fed a HFD for 12 weeks while being injected with Ad-CFTR or Ad-LacZ via tail vein. Western blotting results showed that injection of Ad-CFTR restored the decreased expression of CFTR in aorta from HFD-fed apoE^−/−^ mice (Figure 2A,B). Moreover, Oil red O staining revealed a marked reduction in plaque formation in entire aorta and aortic sinus from HFD-fed apoE^−/−^ mice treated with Ad-CFTR in comparison with mice treated with Ad-LacZ (Figure 2C–E). In addition, overexpression of CFTR exhibited increased relative content of collagen and smooth muscle cells in atherosclerotic plaque, as assessed by quantitative immunostaining, indicating remodeling of plaques toward a more stable lesion phenotype (Figure 2F,G). However, the necrotic regions were profoundly decreased in aortic sinus from Ad-CFTR-treated apoE^−/−^ mice (Figure 2H). These data suggest that up-regulation of CFTR increases plaque stability and attenuates the development of atherosclerosis.

### CFTR reduced vascular inflammation and promoted M2 macrophage polarization during atherogenesis

**Figure 3:**
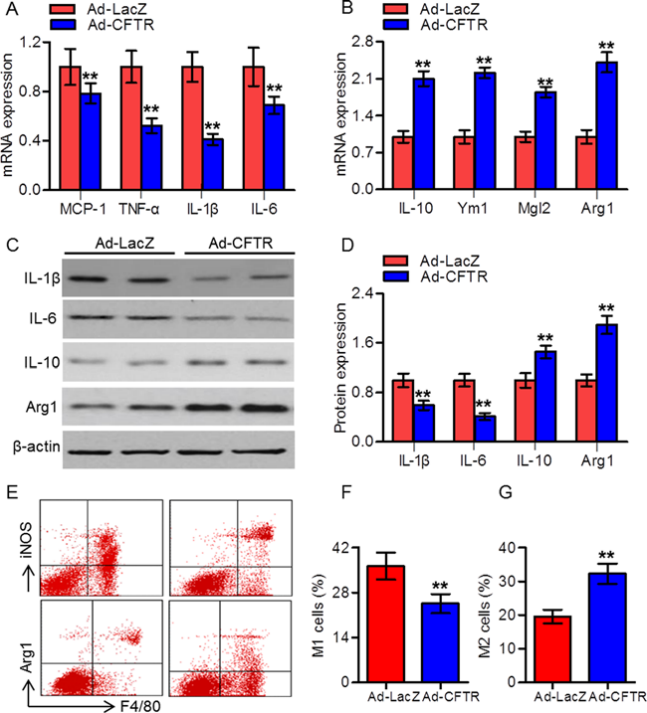
CFTR reduced inflammatory cytokines in aorta and peritoneal macrophages from atherosclerotic mice (**A,B**) RT-PCR analysis for the mRNA expression of MCP-1, TNF-α, IL-1β, and IL-6 (A), and IL-10, Ym1, Mgl2, and Arg1 (B) in aorta from HFD-fed apoE^−/−^ mice treated with Ad-LacZ or Ad-CFTR. **P<0.01 compared with HFD + Ad-LacZ, *n*=6 in each group. (**C**) The protein expressions of IL-1β, IL-6, IL-10, and Arg1 in peritoneal macrophages were determined by Western blotting. (**D**) Quantitation of these protein expressions. **P<0.01 compared with HFD + Ad-LacZ, *n*=6 in each group. (**E**) CFTR up-regulation reduced the proportion of iNOS-positive cells (M1 phenotype) and shifted the macrophage phenotype to Arg1-positive cells (M2 phenotype) compared with Ad-Lacz group. (**F**) Percentage of M1 macrophages. (**G**) Percentage of M2 macrophages. **P<0.01 compared with HFD + Ad-LacZ, *n*=6 in each group.

Inflammation plays an important role in the development of atherosclerosis [4]. We determined the mRNA expression of proinflammatory cytokines in aorta. The results showed that CFTR up-regulation significantly decreased *MCP-1, TNF-α, IL-1β*, and *IL-6* mRNA expression in aorta from HFD-fed apoE^−/−^ mice (Figure 3A). However, the mRNA expression of anti-inflammatory M2 macrophage markers IL-10, Ym1, Mgl2, and Arg1 were increased (Figure 3B). Similar tendency of these cytokines were observed in serum detected by ELISA (Supplementary Figure S2). Furthermore, Western blotting analysis revealed that the protein expression of IL-1β and IL-6 were reduced in peritoneal macrophages isolated from HFD-fed apoE^−/−^ mice treated with Ad-CFTR, whereas the anti-inflammatory factor IL-10 and Arg1 were increased (Figure 3C,D). To further characterize changes in macrophage polarization, flow cytometry was used to assess the M1 marker iNOS, as well as the M2 marker Arg1. Up-regulation of CFTR displayed elevated local expression of M2 macrophages and reduced expression of M1 macrophages compared with that of Ad-LacZ mice (Figure 3E–G).

### Up-regulation of CFTR inhibited the recruitment of immune cells in atherosclerotic plaque

**Figure 4:**
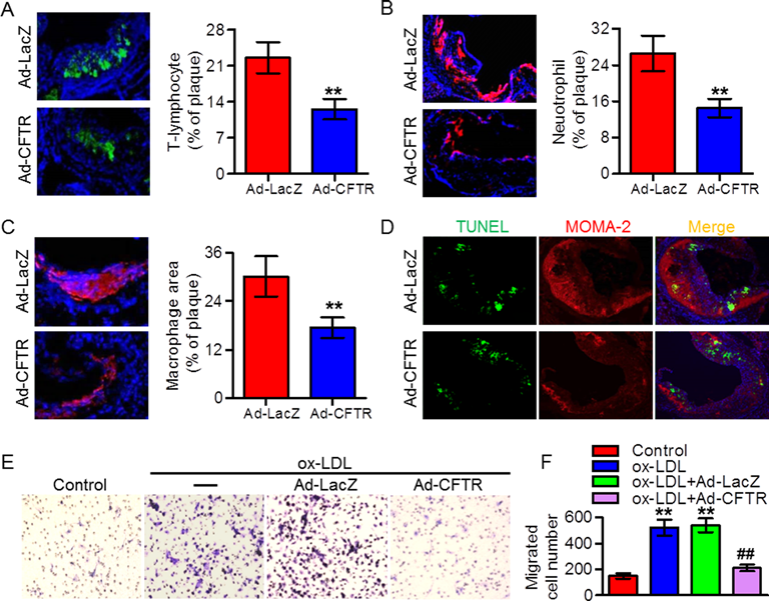
CFTR inhibited immune cells recruitment in atherosclerotic plaque (**A–C**) Laser-scanning confocal microscopy was used to analyze the level of CD3-postive T lymphocytes (A), Ly-6G-positive neutrophils (B), and MOMA-2-positive macrophages (C) in aortic sinus of HFD-fed apoE^−/−^ mice treated with Ad-LacZ or Ad-CFTR, and quantitation of the positive staining area was shown, respectively (400× magnification). **P<0.01 compared with HFD + Ad-LacZ, *n*=6 in each group. (**D**) Representative double immunofluorescence staining of TUNEL (green) and macrophages (red) in aortic sinus (400× magnification). (**E**) Mouse peritoneal macrophages infected with Ad-CFTR or Ad-LacZ for 24 h followed by ox-LDL incubation for another 24 h *in vitro*. The migration of macrophages was assayed (400× magnification). (**F**) The migrated macrophages were counted and quantitated. **P<0.01 compared with control; ^##^*P*<0.01 compared with ox-LDL + Ad-LacZ, *n*=6.

Lesional macrophages can release a number of proinflammatory cytokines and consequently promote immune cells (such as T lymphocytes and neutrophils) infiltration, which is a hallmark of atherogenesis [5,7]. Accordingly, we determined the levels of immune cells infiltration. Treatment of apoE^−/−^ mice with Ad-CFTR decreased the number of CD3-positive T lymphocytes and Ly-6G-positive neutrophils in atherosclerotic plaque compared with that of Ad-LacZ mice (Figure 4A,B). Additionally, we also observed that MOMA-2-positive macrophages content in atherosclerotic plaque was reduced in Ad-CFTR-treated mice (Figure 4C). To further clarify whether the reduced number of macrophages was due to increased apoptotic cells or decreased cell migration, we next examined the effect of CFTR on macrophage survival. As shown in Figure 4D, there were no differences in the TUNEL staining within MOMA-2-positive macrophages. Interestingly, overexpression of CFTR completely abolished ox-LDL-induced migration of mouse peritoneal macrophages (Figure 4E,F), implying that CFTR attenuates macrophage infiltration in atherosclerotic lesion by impairing cell migratory function.

### Activation of NFκB and MAPKs induced by ox-LDL was regulated by CFTR in macrophages

**Figure 5:**
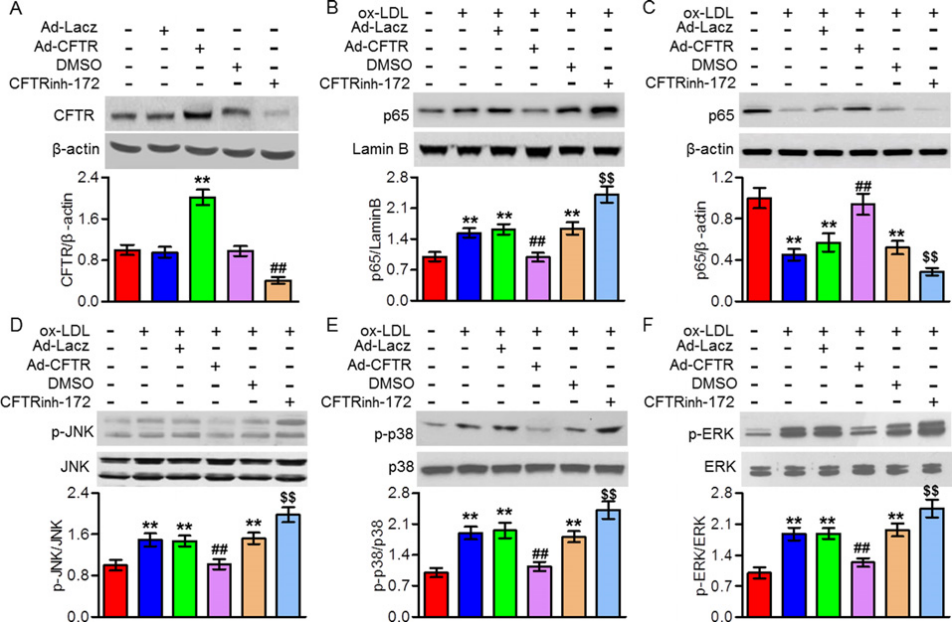
CFTR attenuated ox-LDL-induced NFκB and MAPKs activation (**A**) Mouse peritoneal macrophages were treated with Ad-CFTR (50 multiplicity of infection (MOI)) or CFTRinh-172 (10 μM) for 24 h. Western blotting analysis for the expression of CFTR. **P<0.01 compared with Ad-LacZ; ^##^*P*<0.01 compared with DMSO, *n*=6. (**B–F**) The cells were pretreated with Ad-CFTR or CFTRinh-172 for 24 h, and then stimulated with ox-LDL (80 μg/ml) for another 24 h. Nuclear (B) and cytosol (C) distribution of p65, and JNK (D), p38 (E), and ERK (F) phosphorylation were determined by Western blotting. **P<0.01 compared with control; ^##^*P*<0.01 compared with ox-LDL + Ad-LacZ; ^$$^*P*<0.01 compared with ox-LDL + DMSO, *n*=6.

NFκB and MAPK pathways have been suggested to be associated with CFTR-mediated inflammatory responses [12,20,21]. To test whether the NFκB and MAPKs signaling are involved in CFTR-dependent regulation of inflammation and atherosclerosis, we examined the activities of NFκB and MAPKs in mouse peritoneal macrophages. Transfection of macrophages with Ad-CFTR successfully unregulated CFTR expression, whereas treatment with CFTR inhibitor CFTRinh-172 reduced CFTR expression (Figure 5A). Ox-LDL treatment resulted in a marked p65 nuclear translocation concomitant with phosphorylation of JNK, p38, and ERK, which were all inhibited by CFTR overexpression. However, ox-LDL-induced p65 translocation and JNK, p38 and ERK phosphorylation were further enhanced by pretreatment with CFTRinh-172 (Figure 5B–F). These findings suggest that CFTR attenuated macrophage inflammation during atherosclerosis partially by inhibition of NFκB and MAPKs activation.

### MAPK was the upstream of NFκB in CFTR-dependent macrophage inflammation induced by ox-LDL

**Figure 6:**
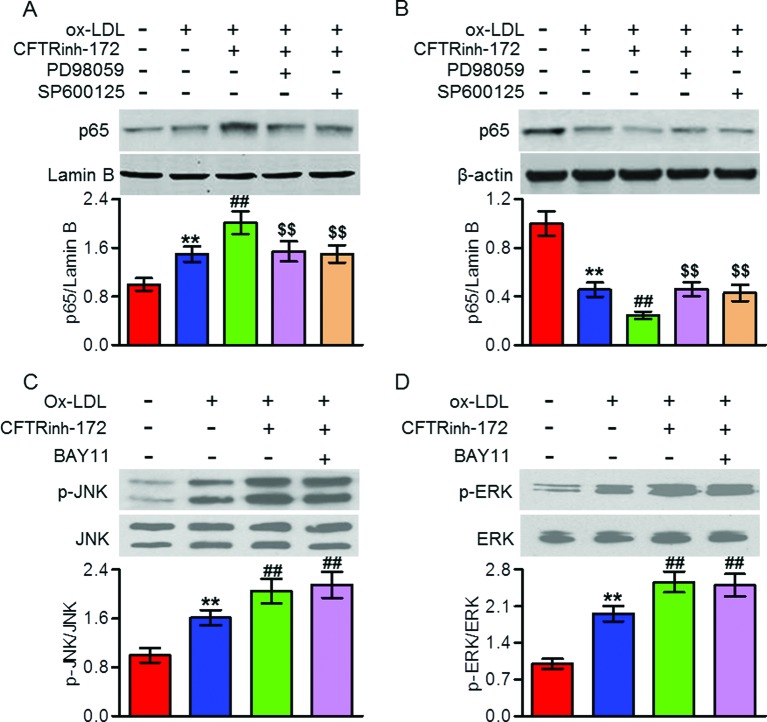
MAPK was the upstream of NFκB signaling in CFTR-mediated macrophages (**A–D**) Peritoneal macrophages were pretreated with CFTRinh-172 (10 μM) for 24 h followed by incubation with ox-LDL (80 μg/ml) for another 24 h in the presence or absence of PD98059 (10 μM), SP600125 (10 μM), or BAY11 (20 μM). Nuclear (A) and cytosol (B) distribution of p65, and JNK (C) and ERK (D) phosphorylation were examined by Western blotting. **P<0.01 compared with control; ^##^*P*<0.01 compared with ox-LDL; ^$$^*P*<0.01 compared with ox-LDL + CFTRinh-172, *n*=6.

To further explore the interaction between NFκB and MAPKs signaling in mediating CFTR-dependent macrophage inflammation, we employed pharmacological inhibitors of ERK, JNK, and NFκB, and then measured their effects on each other’s signaling pathway activation, respectively. Western blotting analysis showed that CFTR knockdown-augmented p65 nuclear translocation was completely inhibited by ERK inhibitor (PD98059) or JNK inhibitor (SP600125) (Figure 6A,B). However, inhibition of NFκB with specific inhibitor BAY11 had no effect on the phosphorylation of JNK and ERK (Figure 6C,D). These results indicate that MAPK is the upstream of NFκB in the CFTR-mediated response.

## Discussion

In the present study, we found that CFTR expression was decreased in atherosclerotic plaque. Up-regulation of CFTR attenuated immune cells infiltration and vascular inflammation as well as atherosclerosis development in apoE^−/−^ mice. Mechanically, CFTR inhibited ox-LDL-induced inflammatory response in macrophages via suppressing NFκB and MAPKs signaling.

Extensive research efforts have sought to characterize the physiological effect of CFTR dysfunction on various tissues throughout the body, amongst which inflammation seems to be closely associated with the underlying mechanism of CF, because CF is initially considered as a hallmark of intestinal and pulmonary inflammation that is caused by CFTR mutation [11,12,22,23]. However, with prominent intestinal and pulmonary disturbances in CF patients, majority of investigators have focussed on epithelial cells inflammation [20–22]. It has been recently shown that CFTR expression was significantly reduced in airway epithelial cells after heat treatment and this down-regulation was responsible for the thermal inhalation injury induced inflammatory responses [20]. Similarly, decrease in endogenous CFTR protein expression was found in VSMCs after hydrogen peroxide treatment [24]. In contrast, CFTR expression was elevated in LPS-induced proinflammatory response in alveolar macrophages [17]. The changes of CFTR expression serve as a causal mediator of the corresponding inflammatory processes. In the present study, we found that CFTR protein was decreased in aorta from HFD-fed apoE^−/−^ mice. Considering that CFTR was colocalized with MOMA-2-positive macrophages in atherosclerotic plaque and ox-LDL had no effect on CFTR expression in endothelial cells and VSMCs, our results suggest that CFTR may participate in macrophages inflammation during the development of atherosclerosis. However, the mechanism by which HFD administration reduces CFTR expression in macrophages, awaits further investigation.

To investigate whether CFTR down-regulation limits the development of atherosclerosis or exerts a proatherogenic role, we used gain-function strategy* in vivo*. The results showed that up-regulation of CFTR decreased Oil red O staining in aorta and aortic sinus from HFD-fed apoE^−/−^ mice. In-line with reduced atherosclerotic lesions, the increase in collagen and VSMC content and decrease in necrotic core were observed in Ad-CFTR-treated apoE^−/−^ mice. These findings demonstrate that CFTR improves plaque stability and attenuates the development of atherosclerosis.

Inflammation and excessive cytokines production are critical events in atherosclerosis, which triggers monocytes and monocyte-derived macrophages recruitment and contributes to the initiation of atherosclerosis [3,6,8]. It has been reported that inflammation is implicated in the pathophysiology of several disorders in CF subjects [9,11,22]. Moreover, CFTR inhibition deteriorated LSP-induced macrophages inflammation and acute lung injury [17]. Consistent with previous studies [9,11,17,22], our results also supported that CFTR plays an important role in vascular inflammation and atherosclerosis. We found that up-regulation of CFTR by adenovirus decreased proinflammatory cytokine expressions and increased anti-inflammatory M2 macrophage markers in aorta and peritoneal macrophages from atherosclerotic apoE^−/−^ mice. Consistently, our results showed that CFTR switched M1 phenotype macrophage to an M2 phenotype. It is important to note that CFTR is also a member of the ATP-binding cassette (ABC) transporter superfamily and is known as ABCC7 [25]. Previous study has indicated that ABC transporters mobilize substrates from the cytoplasm out of the cell with the exception of CFTR [26]. In addition, silencing of CFTR in human alveolar macrophages had no effect on total and free cholesterol levels [27]. These works together with our present study suggest that CFTR prevents atherosclerosis development through inhibiting vascular inflammation rather than influencing lipid transport.

Furthermore, our results consistently revealed obvious reduction in the infiltration of inflammatory cells, such as T lymphocytes, neutrophils, and macrophages. The decrease in macrophages recruitment may be not due to increased cell apoptosis, because CFTR overexpression had no effect on the percentage of TUNEL- and MOMA-2-positive macrophages. Indeed, results of the *in vitro* matrigel invasion model showed that ox-LDL-induced macrophage migration was remarkably inhibited after CFTR overexpression, further supporting that the decreased macrophages infiltration is resulted from inhibiting cell migration but not affecting cell survival. In contrast, a recent study indicated that down-regulation of CFTR expression was associated with endoplasmic reticulum stress and liver cell apoptosis [28]. Moreover, reported by Xu et al. [27] showed that decreased CFTR expression was also involved in alveolar macrophage apoptosis. Presumably, the discrepancy is probably related to: (i) apoptosis may have different roles in different types of cells; (ii) the level of apoptosis is different under different stimulations even in the same cell type; and (iii) the data from *in vitro* is not fully representative as that *in vivo*.

It has been reported that CFTR can regulate the activities of NFκB and MAPKs signaling pathways [12,20,29,30]. Chen et al. [29] demonstrated that NFκB was abnormally activated in CF airway epithelial cells. Moreover, lack of CFTR has also been shown to be abnormally activated MAPKs [12,30]. Meanwhile, these signaling pathways are well known to play important roles in regulating inflammation. However, it is not yet known which is involved in CFTR-regulated vascular inflammation and atherosclerosis. In the current study, we found that overexpression of CFTR inhibited ox-LDL-induced NFκB nuclear translocation and JNK, p38 and ERK phosphorylation in peritoneal macrophages, whereas CFTR inhibition produced the opposite effects. The results indicate that CFTR attenuates vascular inflammation by inhibiting NFκB and MAPKs activity. Interestingly, consistent with a recent study suggesting that MAPKs was the upstream of NFκB in CFTR-regulated airway inflammation [20], our data showed that inhibition of MAPKs abrogated CFTR down-regulation induced NFκB nuclear translocation, whereas NFκB inhibition failed to block MAPKs activation, suggesting NFκB is mediated by MAPKs signaling in this process.

In conclusion, our findings reveal a critical role of CFTR in vascular inflammation and the development of atherosclerotic lesions. The present study suggests that CFTR could be an attractive therapeutic target for the resolution of inflammation and atherosclerosis.

## Abbreviations

α-SMA: α-smooth muscle actin
ABC: ATP-binding cassette
Ad-CFTR: adenovirus encoding CFTR
Ad-LacZ: adenovirus expressing LacZ
apoE^−/−^: apolipoprotein E-deficient
CF: cystic fibrosis
CFTR: CF transmembrane conductance regulator
DMEM: Dulbecco’s modified Eagle’s medium
HFD: high-fat diet
HRP: horseradish peroxidase
IL-1β: interleukin 1β
IL-6: interleukin 6
IL-10: interleukin 10
LPS: lipopolysaccharide
MAEC: mouse aortic endothelial cell
MASMC: mouse aortic smooth muscle cell
MCP-1: monocyte chemoattractant protein
ND: normal diet
ox-LDL: oxidized low density lipoprotein
TNF-α: tumor necrosis factor α
TUNEL: Terminal-deoxynucleoitidyl-mediated dUTP nick end labeling
VSMC: vascular smooth vascular cells
